# Different modalities for treatment of recurrent aphthous stomatitis. A Randomized clinical trial

**DOI:** 10.4317/jced.52877

**Published:** 2016-12-01

**Authors:** Sherine A. Nasry, Hanaa M. El Shenawy, Dina Mostafa, Nagwa M. Ammar

**Affiliations:** 1 Professor. Department of Oral Surgery and Medicine, National Research Centre, Giza, Egypt; 2 Associate Professor. Department of Pharmaceutical Technology, National Research Centre, Giza, Egypt; 3 Professor. Department of Pharmacognosy, National Research Centre, Giza, Egypt

## Abstract

**Background:**

The underlying etiology of recurrent aphthous stomatitis (RAS) is unclear and treatment aims to provide symptomatic and faster relief. This study compared the efficacy of diode laser, a herbal combination of Acacia nilotica and Licorice (A and L) and Amlexanox in the management of RAS.

**Material and Methods:**

Sixty patients with minor aphthae were selected and randomly divided into four groups of 15 each. Group I and II received adhesive preparations of a herbal mixture of A and L and a 2 mg Amlexanox paste respectively, group III received diode laser and the fourth group (control) used a placebo. Ulcer size, pain score were recorded on days 1, 2 and 5.

**Results:**

Laser group showed the statistically highest mean percentage (%) of reduction in pain scores and ulcer size than the other groups. The mean % of reduction in pain scores was 43.3+20.0 at day 2 and 67.8+21.5 % at day 5 in the laser group while Amlexanox group demonstrated a 29.8 +11.3 and 61.9+24.5 mean % of reduction in pain scores at day 2 and 5 respectively. A and L group showed a lower mean % of reduction in pain scores than laser and Amlexanox groups with a 22.2+10.5 and 43.4+15.8 mean % reduction in pain scores at day 2 and day 5 respectively. Similarly the highest mean % of reduction in ulcer size was seen in the laser group being 52.7+19.8 at day 2 and 85.1+22.0 at day 5, while it was 48.1+16.5 at day 2 and 77.8+28.7 at day 5 in the Amlexanox group and 42.0+11.5 at day 2 and 63.0+20.5 at day 5 in the A and L group.

**Conclusions:**

All treatment modalities reduced pain and ulcer size than placebo group. Laser therapy demonstrated the highest percentage of reduction of pain score and ulcer size.

** Key words:**Aphthous stomatitis, laser, herbal plants, Acacia nilotica, Licorice.

## Introduction

Recurrent aphthous stomatitis (RAS) is the most common ulcerative disease of the oral mucosa affecting as high as 15-25 % of the general population worldwide (1). It is a multifactorial disease and several predisposing and risk factors have been implicated in its pathogenesis. Due to uncertain etiology and unpredictable course of the disease, there is no definitive treatment for RAS (2). Treatment strategies therefore aim at relieving pain, promoting healing and preventing secondary infection (3). In severe forms of RAS, systemic agents such as colchicine, dapsone and corticosteroids may be administered to control the symptoms. However most of these therapies are associated with side-effects or unwanted reactions (4). Several topical agents are available for symptom relief in less severe forms of RAS. These include antibacterial, anti-inflammatory, anti-histaminic agents, as well as analgesics, local anesthetics and glucocorticoids (5,6).

Amlexanox 5%, a topical anti-inflammatory and anti-allergic drug was found to play a significant role in the management of minor aphthous ulcers. Amlexanox potentially inhibits the formation and release of histamine and leukotrienes from mast cells, neutrophils, and mononuclear cells. It has been shown to accelerate healing of aphthae and decrease pain, erythema and size of the lesion (7,8). Amlexanox oral paste has been specifically formulated to adhere to the oral mucosa, thus limiting the likelihood of the drug being rubbed or rinsed away with saliva.

On the other hand laser therapy constitutes an alternative therapy to oral diseases that present with pain and inflammatory reactions and that require tissue regeneration, since a laser provides better anti-inflammatory responses with edema reduction, pain reduction and cellular bio-stimulation (9). Apart from RAS, Low level laser therapy (LLLT) has been used in treatment of other mucosal ulcers as lichen planus, pemphigus vulgaris, mucous membrane pemphigoid, etc... However despite publishing a large number of studies about LLLT in oral ulcers, treatment protocols have been conflicting (10).

The use of natural products, including medicinal plant preparations for pain reduction and shortening of healing time of oral aphthous ulcers are gaining more attention due to their decreased side effects and drug resistance (2).

Acacia nilotica (A), commonly known as Indian gum Arabic tree is rich in flavonoids, simple phenolics, saponins, quinines, tannins, coumarins and polysaccharides (11) and is used in the treatment of many diseases In India and Africa it is customarily for treating cancers, and the plant has been also reported to be used in treatment of dental pain (12-14). Extracts of A was found to exhibit an antifungal property against candida albicans and also inhibited *S. mutans* (15). A was found to be the most active plant against bacteria as well as fungal pathogens when compared with other medicinal plants (16).

Licorice (L), the name given to the roots and stolons of Glycyrrhiza species, has been used since ancient times as a traditional herbal remedy (17). L contains several classes of secondary metabolites that have numerous human health benefits and many studies have demonstrated both licorice and its bioactive ingredients to possess potential beneficial effects in several oral diseases which are attributed mainly to the anti-adherence and anti-inflammatory properties of the L compounds (18,19). L used in treating gastric ulcers increased blood supply and mucous secretion thus aiding the healing of ulcerated mucosa (20).

Medicinal plants have a great capability for displaying a synergistic action owing to their multi-component nature (21). Hence combination of both the antibacterial effect of A and the anti-inflammatory effect of L extracts may present a probable synergism that might accelerate the healing of RAS.

The aim of this study was therefore to compare the efficacy of three different treatment modalities: diode laser, a herbal combination of A and L and Amlexanox in the management of RAS.

## Material and Methods

-Plant material

Dried roots and rhizomes of *Glycyrrhiza glabra* L., Family Leguminosae and pods of *Acacia nilotica* LwilldFamily Leguminosae were purchased from a local market in Cairo, Egypt and authenticated by Dr. Abdel Haleem AbdelMotagaly, Dept. of Flora, the Agricultural Museum, Dokki, Giza, Egypt. The plants were crushed and grinded into suitable size for phytochemical study.

-Preparation of biologically active fractions and identifications of compounds

Extraction, isolation and identification of the natural compounds from the two plants were carried out separately *A. nilotica* and *Glycyrrhiza glabra* were extracted separately using solvents of increasing polarity in the following order: petroleumether, ether, chloroform, methanol, and water. These extracts were evaporated to dryness under vacuum at 40°C, lyophilized, and saved for pharmaceutical preparation. The collected polar extracts were subjected to phytochemical investigation using different chromatographic and spectral procedures as; PC, CC, TLC, Preparative HPLC, LC/MS, High field NMR, 1H-NMR, 13C-NMR,HMBC, HMQC, H1H1-COSY, and ESI-MS.

-Preparation of Herbal Adhesive Paste

The adhesive paste was dispensed containing both A and L dry extracts, each at 1% composition forming a total of 2% of the herbal paste. Gelatin, Polyethylene glycol 20000, glycerine, Polyethylene glycol 400, herbal extracts. Tween 20 and distilled water were mixed after weighing appropriate amounts and heated at 70°C till complete dissolution of the components. Similarly, sodium carboxy methyl cellulose and pectin were dispersed in liquid paraffin, mixed well and heated at 70°C. The oily portion was then poured on the aqueous portion with continuous stirring at 70°C till complete homogeneous blending. The formulation was removed from the hot plate and left to cool to room temperature before application by patients for treatment of dental lesions.

-Patients and Methods:

A total of 60 patients, females (62%) and males (38%) with mean age 28.5 years (19-40 years) and a current history of RAS were recruited from the outpatient clinic of Oral Medicine and Periodontology, Oral Diagnosis and Radiology department, Faculty of Dentistry, Ain Shams University. The study has been carried out in accordance with The Code of Ethics of the World Medical Association (Declaration of Helsinki) for experiments involving humans. Before entering the study, eligible subjects were informed regarding the purpose of this study and provided a signed consent to participate in this study. The protocol was approved by Ein Shams University’s Ethical Committee. Inclusion criteria included subjects with a history of at least two confirmed episodes of RAS during a 3-6 months untreated baseline period. Exclusion criteria included pregnant females, smokers or those suffering from psychological disturbances, inflammatory and allergic conditions, or those with special syndrome where RAS is one of its symptoms (e.g. Behcet’s syndrome); those with aphthous lesions older than 4 days, and patients that have received any other treatment for the last 4 weeks.

-Study design

Subjects satisfying the inclusion criteria were assigned randomly to one of the four-treatment groups: group I received adhesive pastes of A and L, group II received adhesive oral tablets of 2mg Amlexanox *, group III received diode laser irradiation and group IV assigned as the control group received a placebo adhesive tablet. Patients of group I, II and IV were instructed to apply the medication on the lesion q.i.d. after drying it with a small sterile cotton pad and refrain from eating at least for 30 min after the drug application. Treatment continued till for 5 days. A 970 nm diode lasers was used for the treatment of RAS in a non-contact mode. A 320 micron fiber optic tip diameter was used to deliver the laser beam; the initiation was done on an articulating paper. Then diode Siro laser ** was started at a defocused mode from the lesion (5-8 mm) and advanced slowly towards the area ending up 2-3mm away and continuously moving from the periphery of the lesion to the center “painting “ the entire area and moving away from the lesion if the patient feels warmth. The setting was put at 0.8w for 30-45 seconds with a refractory period of 15-20 seconds between laser “passes” to allow for tissue relaxation. The area was rubbed gently with a wet gloved finger to determine if a decrease in pain was felt by the patient. A 2nd or a 3rd laser pass was occasionally needed to decrease the pain with a maximum time of three minutes total irradiation time. The laser treatment consisted of one sitting which started at day 0. Each sitting consisted of a maximum of four sessions of applications.

*Aphthatab, EVA pharma,. Cairo, Egypt.

 **970nm SIRO Laser Advance class IIIb, SIRONA The Dental company, Germany.

After each pass, the area was checked with palpation. The patients were asked to refrain from using any medications over the next 5 days. The pain scores and sizes of the ulcers were evaluated on treatment days 0, 2 and 5. Pain was recorded using the visual analog scale (VAS), from 1-10 with 10 being most severe, and subjects were instructed to mark the number representing their level of perceived pain. The size of the ulcer was measured by a single oral specialist who was blinded to the subject’s status using a William’s graduated periodontal probe.

-Statistical analysis

Numerical data were explored for normality by checking the distribution of data, calculating the mean and median values as well as using tests of normality (Kolmogorov-Smirnov and Shapiro-Wilk tests). All data showed non-parametric distribution. Data were presented as mean and standard deviation (SD) values. Kruskal-Wallis test was used to compare between the four groups. Mann-Whitney U test was used to for pair-wise comparisons when Kruskal-Wallis test is significant. Bonferroni’s correction was applied for the pair-wise comparisons.The significance level was set at *P* ≤ 0.05. Statistical analysis was performed with IBM® SPSS® Statistics Version 20 for Windows.

## Results

-Pain score

**Figure 1:**
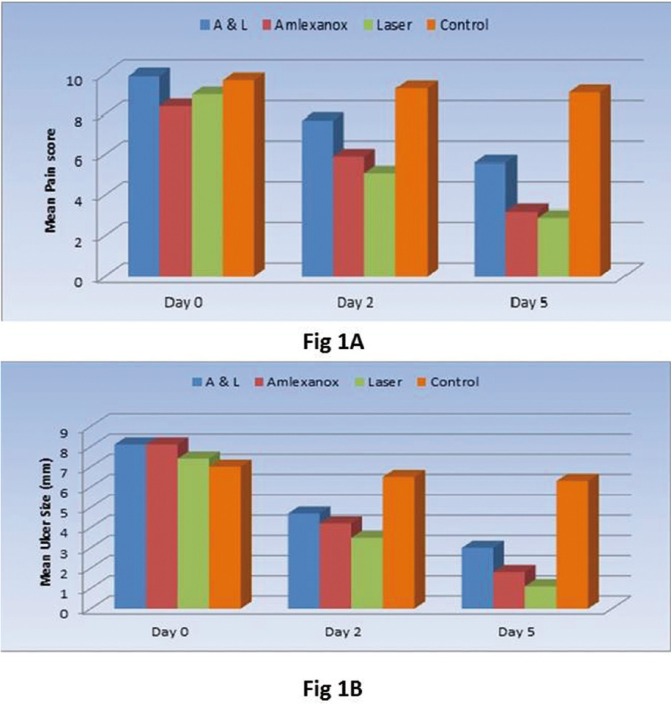
A,B) Mean pain score and mean ulcer size before and after treatment in the four groups.

At day 2, and 5 control group showed the statistically significantly highest mean pain score which was (9.3 +0.5) at day 2 and 9.1+0.7 at day 5. No statistically significant difference was observed between the three treatment groups at day 2. (*p*=0.010), where mean pain scores were 7.7+1.1, 5.9+1.1, and 5.1+1.2 for A and L, Amlexanox and Laser groups respectively. At day 5 both Amlexanox and Laser groups demonstrated statistically significantly lower mean pain scores than A and L group (*p* < 0.001), where the mean pain score results for the Amlexanox group was 3.2+1.6 and for the laser group 2.9 +1.1 while that of A and L group was 5.6+1.3 ( Table 1, Fig. 1A).

**Table 1:**
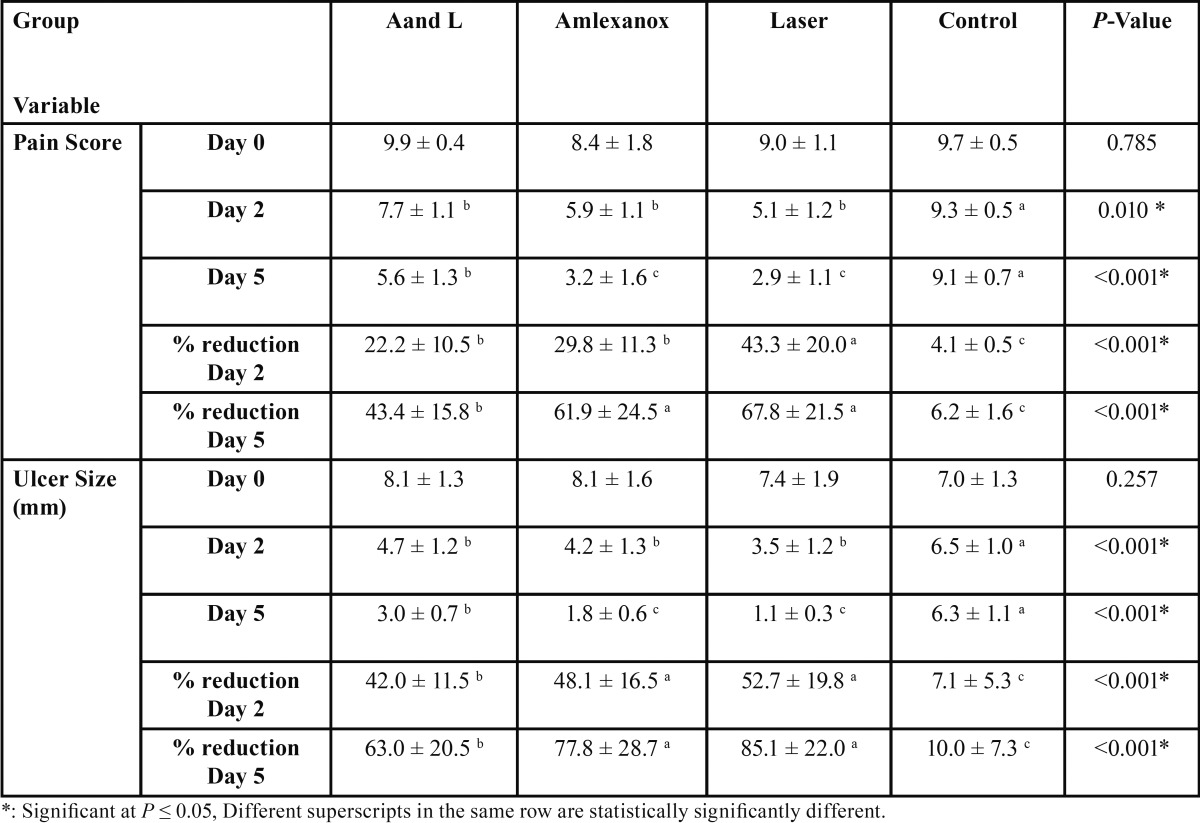
Mean ± standard deviation (SD) values and results of comparison between different parameters in the four groups.

-Ulcer size

Similar to the pain score, control group showed the statistically significantly highest mean ulcer size in both days 2 and 5 with a mean ulcer size of 6.5+1.0 at day 2 and 6.3+1.1 at day 5. Statistical results demonstrated no statistical difference between all three treatment modalities at day 2; where the mean ulcer size recorded 4.7+1.2, 4.2+1.3 and 3.5+1.2 for A and L, Amlexanox and Laser groups respectively. At day 5, A and L group showed statistically significantly lower mean ulcer size than the control group (*p*<0.001), while Amlexanox and Laser groups; both showed the lowest mean ulcer sizes with no statistically significant difference between them. The mean ulcer size was 3.0+0.7, 1.8+0.6 and 1.1+0.3 for A and L, Amlexanox and Laser groups respectively ( Table 1, Fig. 1B).

**Figure 2:**
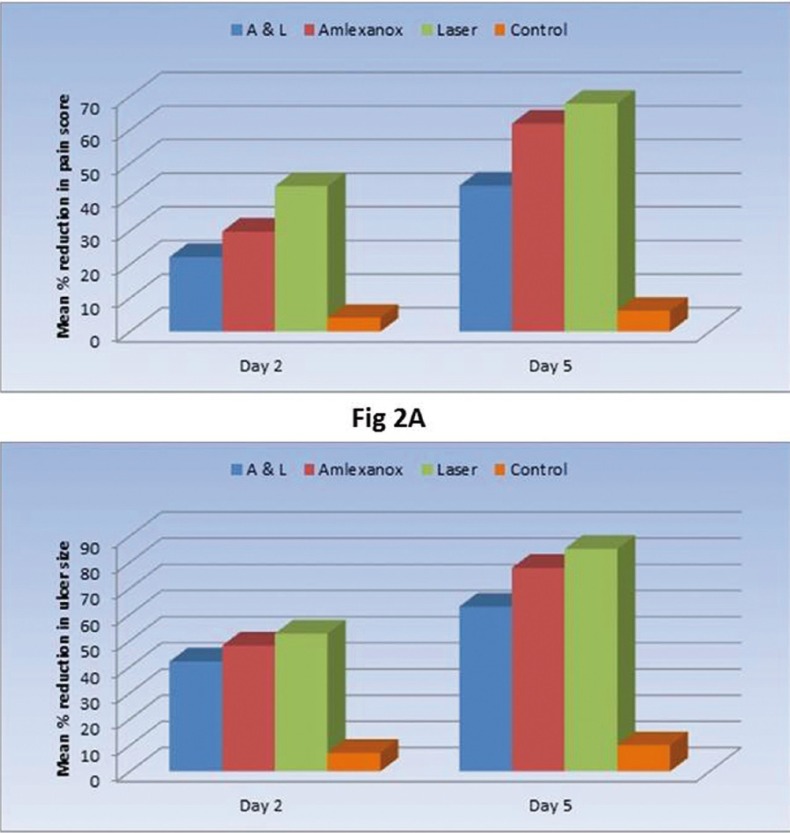
A,B) Mean % of reduction in pain score and ulcer size after treatment in the four groups.

Regarding the percentage of reduction, Laser group showed the statistically significantly highest mean percentage (%) of reduction in pain scores and ulcer size than the other groups. The mean % of reduction in pain scores was 43.3+20.0 (at day 2) and 67.8+21.5 % (at day 5) in the laser group in comparison to Amlexanox group which demonstrated a 29.8+11.3 and 61.9+24.5 mean % of reduction in pain scores at day 2 and 5 respectively. A and L group showed a lower mean % of reduction in pain scores than laser and Amlexanox groups with a 22.2+10.5 and 43.4+15.8 mean % reduction in pain scores at day 2 and day 5 respectively. Similarly the highest mean % of reduction in ulcer size was seen in the laser group being 52.7+19.8 at day 2 and 85.1+22.0 at day 5, while it was 48.1+16.5 at day 2 and 77.8+28.7 at day 5 in the Amlexanox group and 42.0+11.5 at day 2 and 63.0+20.5 at day 5 in the A and L group. The control group showed the statistically significantly lowest mean % reduction in pain and ulcer size scores ( Table 1, Figs. 2 A,B).

## Discussion

The multifactorial etiology of RAS and lack of effective treatment modality makes the therapeutic aim to provide symptomatic relief for the patient, aiming mainly at decreasing pain, size, and number of ulcers (22). Not all treatment modalities are successful in achieving all these goals, and therefore it is important to evaluate any clinical trial to determine if a medication should be recommended for widespread use (1).

In the present study a comparison between the efficacies of diode laser, a mixture of A and L, Amlexanox on reduction of pain and lesion size in RAS was performed. The frequency of recurrence was not investigated in our study. Although some treatments were successful in reducing the frequency of recurrence in RAS, no study could come into agreement with respect to reducing the recurrence rate of aphthous ulcers (23). No routine laboratory procedures were necessary since history and clinical examination of the lesions were characteristic of RAS (24).

The reason behind choosing diode laser counterparts is the many advantageous this type has over high powered laser. Diode lasers do not cause thermal injury to the tissues, are affordable, easy to use, with superior electrical and optical efficiencies (25). One W was used in this study, because all tissues reactions in aphthous ulcerations occur in the epithelium negating the use of high power laser treatment. Using higher doses tend to decrease cellular proliferation thus slowing the healing process (26). A refractory period of 15-20 seconds between laser “passes” was allowed to give the chance for the tissue to cool down (27).

When statistically comparing the pain and ulcer size between the groups, all three study group demonstrated reduction in pain and ulcer size than the control (placebo) group at all periods. The control group also demonstrated reduction in pain and ulcer size throughout the different study periods. This could be explained by the patients’ feeling of being treated giving them a sense of relief, or the placebo forming a film covering the ulcer thus providing a protective mechanical effect of the placebo possibly forming a film covering the ulcer and providing some soothing effect. It could be also due to the nature of the ulcer itself, being self-limiting (28).

Laser group demonstrated the lowest mean percentage of reduction in both pain scores and ulcer size at day 2 and 5, with a statistically significant difference between laser group and both A and L and control group. The effect of Amlexanox on pain and ulcer size reduction was close to that of laser with no statistical significant difference observed between both groups except at day 2 where laser demonstrated a statistically significant lower percentage of pain reduction denoting the faster effect of laser in alleviating pain in RAS patients.

It was proposed that LLLT accelerates wound healing and reduces pain by stimulating collagen production, altering DNA synthesis and improving the function of neurological tissue. In two case reports LLLT provided instant pain relief and rapid decrease in ulcer size which is in accordance with the results of the present study (25). De Souza *et al.* (27) revealed reduction in pain in the same session after laser treatment and total regression of the lesion in 4 days in comparison to corticosteroid therapy where total regression occurred in 5-7 days.

Many studies demonstrated improvement in healing with reduction of pain, size, duration and recurrence of RAS when Amlexanox paste was applied. Authors attributed this effect to Amlexanox causing inhibition of release of histamines and leukotrienes from mast cells and neutrophils and the release of inflammatory cytokine IL-1 beta, thus increasing the vascular permeability and aiding in the healing of the ulcer (8,24). Since Amlexanox oral paste is easily available and affordable, easy to carry and demonstrated no significant unfavorable taste, some patients might prefer it over laser which requires a more precise and lengthy procedure.

In the present study, the group using A and L demonstrated better results than the placebo group, however the percentage of reduction in both pain scores and ulcer size in the A and L group was less than that of the laser and Amlexanox groups. The use of herbal extracts either individually or in a combination of more than one extract has shown to be effective in treating many diseases including oral ulcers (15), which is in accordance with the results of the present study. However while some studies using natural medications in RAS treatment demonstrated significant improvement in pain, and reduction of ulcer size and duration of ulcers other results proved insignificant when the efficacy of herbal medication on ulcer size and healing time was compared with placebo results (28,29).

It can therefore be concluded that all three treatment modalities reduced pain and ulcer size in comparison with placebo group, however RAS treatment using diode laser demonstrated the highest percentage of reduction in pain scores and ulcer size. Thus it is safe to assume that the choice of treatment modality might depend on the case itself. Since aphthous ulcers are recurrent, studies on a larger no of subjects and for a longer term may be needed for more beneficial results.

## References

[1] Bhat S, Sujatha D (2013). A clinical evaluation of 5% amlexanox oral paste in the treatment of minor recurrent aphthous ulcers and comparison with the placebo paste: a randomized, vehicle controlled, parallel, single center clinical trial. Indian J Dent Res.

[2] Ghalayani P, Zolfaghary B, Farhad AR, Tavangar A, Soleymani B (2013). The efficacy of Punicagranatum extract in the management of recurrent aphthous stomatitis. J Res Pharm Pract.

[3] Preeti L, Magesh K, Rajkumar K, Karthik R (2011). Recurrent aphthous stomatitis. J Oral Maxillofac Pathol.

[4] Scully C, Gorsky M, Lozada-Nur F (2003). The diagnosis and management of recurrent aphthous stomatitis: A consensus approach. J Am Dent Assoc.

[5] MacPhail L (1997). Topical and systemic therapy for recurrent aphthous stomatitis. Semin Cutan Med Surg.

[6] Barrons RW (2001). Treatment strategies for recurrent oral aphthous ulcers. Am J Health Syst Pharm.

[7] Bell J (2005). Amlexanox for the treatment of recurrent aphthous ulcers. Clin Drug Investig.

[8] Meng W, Dong Y, Liu J, Wang Z, Zhong X, Chen R (2009). A clinical evaluation of amlexanox oral adhesive pellicles in the treatment of recurrent aphthous stomatitis and comparison with amlexanox oral tablets: a randomized, placebo controlled, blinded, multicenter clinical trial. Trials.

[9] Aggarwal H, Singh MP, Nahar P, Mathur H, Sowmya GV (2014). Efficacy of Low-Level Laser Therapy in Treatment of Recurrent Aphthous Ulcers – A sham controlled split mouth follow up study. J Clin Diagn Res.

[10] Basirat M (2012). The Effects of the Low Power Lasers in the Healing of the Oral Ulcers. J Lasers Med Sci.

[11] Sharma AK, Kumar A, Yadav SK, Rahal A (2014). Studies on Antimicrobial and Immunomodulatory Effects of Hot Aqueous Extract of Acacia nilotica L. Leaves against Common Veterinary Pathogens. Vet Med Int.

[12] Ali A, Akhtar N, Khan BA, Khan MA, Rasul A, Zaman SU (2012). Acacia nilotica: a plant of multipurpose medicinal uses. J Med Plants Res.

[13] Auwal MS, Saka S, Mairiga IA, Sanda KA, Shuaibu A, Ibrahim A (2014). Preliminary phytochemical and elemental analysis of aqueous and fractionated pod extracts of Acacia nilotica (Thorn mimosa). Vet Res Forum.

[14] Kalaivani T, Mathew L (2010). Free radical scavenging activity from leaves of Acacia nilotica (L.) Wild. Ex Delile, an Indian medicinal tree. Food Chem Toxicol.

[15] Chandra Shekar BR, Nagarajappa R, Suma S, Thakur R (2015). Herbal extracts in oral health care - A review of the current scenario and its future needs. Pharmacogn Rev.

[16] Khan R, Islam B, Akram M, Shakil S, Ahmed A, Ali M (2009). Antimicrobial activity of five herbal extracts against multi drug resistant (MDR) strains of bacteria and fungus of clinical origin. Molecules.

[17] Davis EA, Morris DJ (1991). Medicinal uses of licorice through the millennia: the good and plenty of it. Mol Cell Endocrinol.

[18] La VD, Tanabe S, Bergeron C, Gafner S, Grenier D (2011). Modulation of matrix metalloproteinase and cytokine production by licorice isolates licoricidin and licorisoflavan A: potential therapeutic approach for periodontitis. J Periodontol.

[19] Messier C, Epifano F, Genovese S, Grenier D (2012). Licorice and its potential beneficial effects in common oro-dental diseases. Oral Dis.

[20] Goso Y, Ogata Y, Ishihara K, Hotta K (1996). Effects of traditional herbal medicine on gastric mucin against ethanol-induce gastric injury in rats. Comp Biochem Physiol C Pharmacol Toxicol Endocrinol.

[21] Yang Y, Zhang Z, Li S, Ye X, Li X, He K (2014). Synergy effects of herb extracts: pharmacokinetics and pharmacodynamicbasis. Fitoterapia.

[22] Deshmukh RA, Bagewadi AS (2014). Comparison of effectiveness of curcumin with triamcinolone acetonide in the gel form in treatment of minor recurrent aphthous stomatitis: A randomized clinical trial. Int J Pharm Investig.

[23] Jurge S, Kuffer R, Scully C, Porter SR (2006). Mucosal disease series. Number VI. Recurrent aphthous stomatitis. Oral Dis.

[24] Natah SS, Konttinen YT, Enattah NS, Ashammakhi N, Sharkey KA, Häyrinen-Immonen R (2004). Recurrent aphthous ulcers today: a review of the growing knowledge. Int J Oral Maxillofac Surg.

[25] Anand V, Gulati M, Govila V, Anand B (2013). Low level laser therapy in the treatment of aphthous ulcer. Indian J Dent Res.

[26] Sattayut S, Trivibulwanich J, Pipithirunkarn N, Danvirutai N (2013). A clinical efficacy of using CO2 laser irradiating to transparent gel on aphthous stomatitis patients. Laser Ther.

[27] De Souza TO, Martins MA, Bussadori SK, Fernandes KP, Tanji EY, Mesquita-Ferrari RA (2010). Clinical evaluation of low-level laser treatment for recurring aphthous stomatitis. Photomed Laser Surg.

[28] Moghadamnia AA, Motallebnejad M, Khanian M (2009). The efficacy of the bioadhesive patches containing licorice extract in the management of recurrent aphthous stomatitis. Phytother Res.

[29] Haghpanah P, Moghadamnia AA, Zarghami A, Motallebnejad M (2015). Muco-bioadhesive containing ginger officinale extract in the management of recurrent aphthous stomatitis: A randomized clinical study. Caspian J Intern Med.

